# Transmitted Drug Resistance in the CFAR Network of Integrated Clinical Systems Cohort: Prevalence and Effects on Pre-Therapy CD4 and Viral Load

**DOI:** 10.1371/journal.pone.0021189

**Published:** 2011-06-20

**Authors:** Art F. Y. Poon, Jeannette L. Aldous, W. Christopher Mathews, Mari Kitahata, James S. Kahn, Michael S. Saag, Benigno Rodríguez, Stephen L. Boswell, Simon D. W. Frost, Richard H. Haubrich

**Affiliations:** 1 BC Centre for Excellence in HIV/AIDS, Vancouver, British Columbia, Canada; 2 Department of Medicine, University of California San Diego, San Diego, California, United States of America; 3 University of Washington, Seattle, Washington, United States of America; 4 University of California San Francisco, San Francisco, California, United States of America; 5 University of Alabama Birmingham, Birmingham, Alabama, United States of America; 6 Case Western Reserve University, Cleveland, Ohio, United States of America; 7 Fenway Community Health/Harvard Medical School, Boston, Massachusetts, United States of America; 8 University of Cambridge, Cambridge, United Kingdom; South Texas Veterans Health Care System, United States of America

## Abstract

Human immunodeficiency virus type 1 (HIV-1) genomes often carry one or more mutations associated with drug resistance upon transmission into a therapy-naïve individual. We assessed the prevalence and clinical significance of transmitted drug resistance (TDR) in chronically-infected therapy-naïve patients enrolled in a multi-center cohort in North America. Pre-therapy clinical significance was quantified by plasma viral load (pVL) and CD4+ cell count (CD4) at baseline. Naïve bulk sequences of HIV-1 protease and reverse transcriptase (RT) were screened for resistance mutations as defined by the World Health Organization surveillance list. The overall prevalence of TDR was 14.2%. We used a Bayesian network to identify co-transmission of TDR mutations in clusters associated with specific drugs or drug classes. Aggregate effects of mutations by drug class were estimated by fitting linear models of pVL and CD4 on weighted sums over TDR mutations according to the Stanford HIV Database algorithm. Transmitted resistance to both classes of reverse transcriptase inhibitors was significantly associated with lower CD4, but had opposing effects on pVL. In contrast, position-specific analyses of TDR mutations revealed substantial effects on CD4 and pVL at several residue positions that were being masked in the aggregate analyses, and significant interaction effects as well. Residue positions in RT with predominant effects on CD4 or pVL (D67 and M184) were re-evaluated in causal models using an inverse probability-weighting scheme to address the problem of confounding by other mutations and demographic or risk factors. We found that causal effect estimates of mutations M184V/I (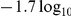 pVL) and D67N/G (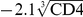 and 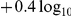 pVL) were compensated by K103N/S and K219Q/E/N/R. As TDR becomes an increasing dilemma in this modern era of highly-active antiretroviral therapy, these results have immediate significance for the clinical management of HIV-1 infections and our understanding of the ongoing adaptation of HIV-1 to human populations.

## Introduction

A substantial fraction (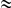
5%–20%) of human immunodeficiency virus type 1 (HIV-1) variants that are transmitted and establish infections in individuals who have never been exposed to antiretroviral drugs (*i.e.*, ARV-naïve) already carry one or more mutations that are associated with resistance to ARVs [1], [2]. Resistance mutations are generally slow to revert in ARV-naïve hosts [3], [4] and can persist at low frequencies in treatment-experienced hosts as well [5]. The pre-existence of resistance mutations can significantly diminish effectiveness of subsequent ARV therapy [6], [7]; for example, transmitted K103N mutations are associated with increased regimen failure and poorer outcomes on non-nucleoside reverse transcriptase inhibitors (NNRTIs) [8]. On the other hand, resistance mutations can also incur a measurable cost with respect to virus replicative capacity *in vitro* [9]–[11] and competitive growth *in vivo* [12]. Within therapy-naïve hosts, the fitness costs of transmitted drug resistance (TDR) mutations may influence the subsequent evolution and population dynamics of HIV-1. With the exception of limiting the choice of initial ARV regimens, however, the direct clinical consequences of TDR mutations in therapy-naïve hosts remain inconclusive [13]–[17].

Our central hypothesis in this study is that the presence of TDR mutations in therapy-naïve hosts has a measurable effect on not only the subsequent rate of growth of the virus population within the host, but also on the rate of depletion of CD4+ T-lymphocytes that are targeted by HIV-1. To evaluate this hypothesis, we assess the relationship between TDR mutations and prognostic clinical markers – CD4+ T-cell count (CD4) and plasma HIV-1 RNA load (pVL) – at baseline using data from the Centers for AIDS Research (CFAR) Network of Integrated Clinical Systems (CNICS), an observational cohort created from a data-sharing collaboration between multiple clinical sites in North America [18]. ‘Baseline’ refers to the measurements of clinical markers upon HIV diagnosis and/or entry into clinical care. It does not correspond to a consistent time point after infection and many patients will have already progressed into the chronic phase of infection. Nevertheless, these data can provide useful insight into the clinical implications of TDR in therapy-naïve hosts.

First, we quantify the effects of TDR mutations at both aggregate and position-specific levels. An aggregate approach sums across mutations occupying different positions in HIV-1 protease and reverse transcriptase, implicitly assuming that every mutation has a similar effect (in both direction and magnitude) on baseline CD4 or pVL. In contrast, a position-specific analysis recognizes the possibility that some TDR mutations will have a disproportionately large effects while others have negligible effects. Furthermore, it enables us to identify compensatory interactions, such that the effect of a specific mutation on CD4 or pVL depends on the presence or absence of other mutations; such interactions are an important aspect of the evolution of drug resistance in HIV-1 [11]. Second, we re-evaluate statistically significant associations of individual TDR mutations in a causal modeling framework, *i.e.*, inverse probability-of-treatment weighting [19]. A statistical association between a treatment (a TDR mutation) and an outcome (baseline CD4 or pVL) in an observational study may be biased if one or more variables, such as other co-transmitted resistance mutations, are not only correlated with the treatment but also affect the outcome [19]. In the presence of such confounding factors, the significant association could be wrongly attributed to a causal effect of the treatment on the outcome. Using statistical procedures that can adjust for confounding by weighting observational data has become an important component of understanding the consequences of drug resistance in HIV [20].

## Methods

### Ethics statement

All patients in the CNICS database have provided informed written consent, as reviewed and approved by the institutional review boards of all participating sites – University of Alabama, Birmingham (UAB); University of California, San Diego (UCSD); University of California, San Francisco (UCSF); University of Washington (UW); Case Western Reserve University (CWRU); and Harvard University (FENWAY) – to have their clinical information used for the purposes of research. Patients were informed on the minimum information being collected, alternatives to participation, and possible uses of the data. In addition, CNICS has received a Certificate of Confidentiality from the National Institutes of Health (NIH).

### Data collection

The study population comprised individuals from 6 CNICS sites: University of Alabama, Birmingham; University of California, San Diego; University of California, San Francisco; University of Washington; Case Western Reserve University; and Harvard University [18]. All patients in the CNICS database have provided informed written consent, as reviewed and approved by the institutional review boards of all participating sites, to have their clinical information used for the purposes of research. HIV-1 *pol* sequences in the region encoding protease and/or part of reverse transcriptase (*i.e.*, the first 246–400 codons of RT) were obtained at each site by conventional Sanger sequencing directly from RT-PCR amplification products from sample extractions of viral RNA. The exact bulk sequencing protocol varied among sites, as some sites used commercial laboratory services (*viz.*, Monogram, Quest, Virco, LabCorp) whereas others used in-house assays based on commercial kits. At the time of this study, 4914 codon or amino acid sequences were available in the CNICS database but many were obtained when patients were on-therapy. Amino acid sequences were reconstituted from reports of residue polymorphisms relative to the HXB2 reference sequence. Sequences were linked to: unique anonymized patient identifiers; CNICS site; sampling date; age; ARV regimens; demographic factors (self-reported gender at baseline, race/ethnicity); HIV risk factors (men who have sex with men, MSM; injection drug use, IDU); baseline plasma HIV RNA titres (pVL, virus/mL plasma); and baseline CD4 cell counts (cells/mL). A patient record was included in this study if: (1) a sequence was sampled before therapy, and (2) CD4 and/or pVL were sampled within 120 days of the sequence sample date. To normalize the distributions of pVL and CD4, we used 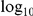 and cube-root transformations [21], respectively. Race and ethnicity records were aggregated into the following groups: non-Hispanic white, non-Hispanic black, Hispanic, and other. Non-random associations between demographic and risk factors were identified using log-linear models.

### Sequence analysis

Sequences were aligned pairwise against the NL4-3 *pol* reference sequence using an implementation of the Gotoh algorithm in HyPhy [22], [23] under default settings. Surveillance drug resistance mutations (SDRMs) were tallied for each amino acid sequence according to the 2009 update of the World Health Organization list of surveillance drug resistance mutations (SDRMs) [24]. Amino acid polymorphisms (*e.g.*, bulk sequence mixtures) were resolved to the resistant residue when applicable.

### Statistical analysis

Resistance scores by drug class (protease inhibitors, PIs; nucleoside and non-nucleoside reverse transcriptase inhibitors, NRTIs/NNRTIs) were calculated according to the Stanford HIV Drug Resistance database algorithm [25]. The Stanford scoring system is a linear predictor of drug resistance phenotypes that works by assigning integer weights to a sequence for encoding resistance-associated amino acid residues at specific positions. Weights (or ‘scores’) for a given sequences were calculated for every drug in a given drug class. We used the highest score of any drug in a drug class as the class-specific score. These quantitative scores, denoted by 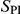, 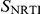 and 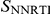 respectively, were used to analyze the aggregate effects of TDR on baseline CD4 and pVL. Aggregate or position-specific effects of TDR on baseline CD4 or pVL were evaluated alongside demographic and risk factors in linear models. In each case we used a bi-directional stepwise algorithm in R (*stepAIC* [26]) to select a ‘best-fitting’ linear model based on the Akaike information criterion (AIC), which penalizes model likelihood by the number of parameters. We seeded the algorithm with the full additive model (without interaction terms) and limited model complexity to second-order interactions. To avoid over-fitting the data from evaluating a large number of alternative models, we averaged parameter estimates and 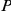 values over an ensemble of models (best +5 next-best) using Akaike weights [27].

To estimate causal effects of individual SDRMs, we used inverse probability-of-treatment weight (IPTW) estimators [19]. The conditional probability 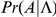 for a given ‘treatment’ variable (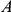) was computed by fitting a logistic model of 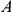 against the set of all potential confounding variables (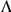). To account for compensatory interactions among mutations, we combined the corresponding presence/absence variables into a single multinomial treatment as suggested to us by Miguel A. Hernán (pers. comm.). For example:
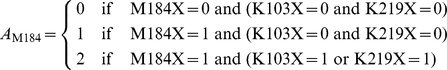
where 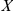 signifies any SDRM residue at that position. Conditional probabilities for multinomial treatments were obtained by fitting a multinomial logistic model with the *multinom* function in the R package *nnet*. Because weighting observations by the inverse of 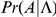 can introduce excessive variability, we used stabilized weights that normalize 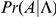 by the marginal probability 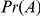, which was estimated by fitting a logistic model containing only the intercept term [19]. The stabilized weights were incorporated into a linear model of baseline CD4 or pVL on treatment 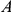 using the *svyglm* function in the R package *survey* [28], which uses an estimating equation-based approach to fitting generalized linear models with robust standard errors. Cases with missing data were omitted from these analyses.

A Bayesian network analysis to detect co-transmission of TDR mutations was carried out using a Markov chain Monte Carlo (MCMC) procedure in HyPhy [29]. A Bayesian network is a compact representation of the joint probability distribution of multiple variables (*i.e.*, the presence/absence of TDR mutations), where ‘joint’ implies that the probability distribution of a variable may be conditional on other variables in the network [30]. In other words , a Bayesian network provides an efficient framework for exploring the statistical interactions among variables. Each variable is represented by a ‘node’ in the network, and connections between nodes (‘edges’) are drawn to indicate that one variable is conditioned on the second. The MCMC procedure obtains a random sample of Bayesian networks from the posterior probability distribution defined by the data. The Markov chain was propagated for 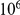 steps, discarding the first half as burn-in and thinning the remaining sample to 100 steps at equal intervals. Edges in the network signifying co-transmission were accepted if they were present in 90% or more of the networks in the sample.

## Results

### Population

The study population was comprised of 14111 individuals with a median baseline CD4 of 300 cells/mL (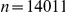) and a median baseline pVL of 24400 copies/mL (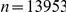). Median year of birth was 1964. The majority of the population self-reported as ‘white’ (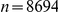). 3725 individuals self-reported as ‘black’, 207 as ‘Asian/Pacific Islander’ and 835 as ‘other’; there were 650 non-respondents. At the time of this study, the CNICS database contained 1585 sequence records that we classified as therapy-naïve. Out of these, 1575 naïve sequences were linked to baseline CD4 and pVL records. 1526 naïve sequences were associated with baseline CD4 records within 120 days of the sequence sampling date and were otherwise censored from subsequent analyses. Likewise, 1516 naïve sequences were associated with baseline pVL within 120 days of sampling and were otherwise censored. The median baseline CD4 and pVL of patients with naïve sequences in this data set were 285 cells/mL and 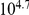 copies/mL, respectively. Composition of the naïve sample with respect to race was significantly associated with gender (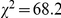, 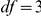, 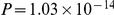); *e.g.*, the sample included a disproportionately greater number of naïve sequences from white males (odds ratio, 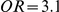) and black females (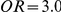). Sample composition by race was also significantly associated with MSM (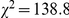, 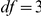, 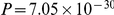), largely because of a greater number of white MSMs than expected by marginal frequencies (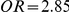).

### Prevalence of transmitted SDRMs

**Figure 1:**
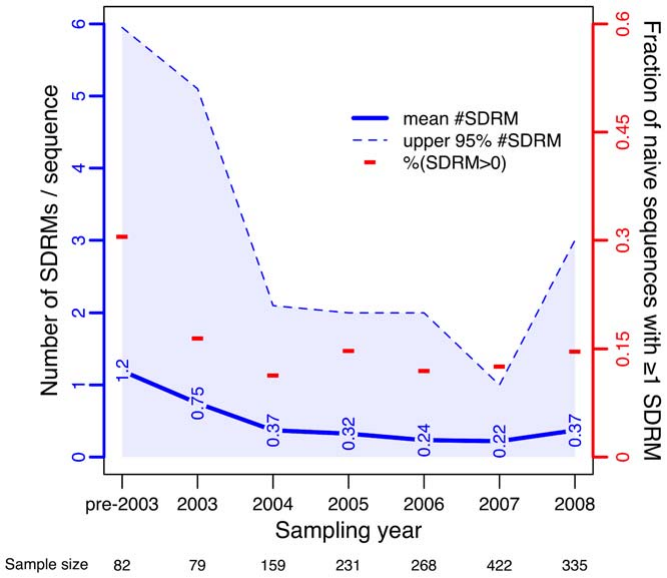
Prevalence of transmitted surveillance drug resistance mutations (SDRMs) over time. Sampling years 1998–2002 were grouped into a single level (‘pre-2003’) to adjust for small sample sizes (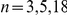, and 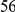, respectively). Sample sizes for the bins are displayed below the 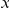-axis. Dashes (**−**) indicate the fraction of naïve sequences in a given sampling period that contain at least one SDRM (right 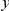-axis). The trend in mean number of SDRMs per naïve sequence (left 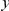-axis) over time is displayed as a solid line annotated with the actual values. The 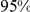 quantile per time point is depicted by a lighter line bounding a shaded region to illustrate the trend in the distributions (all medians were zero).

**Figure 2:**
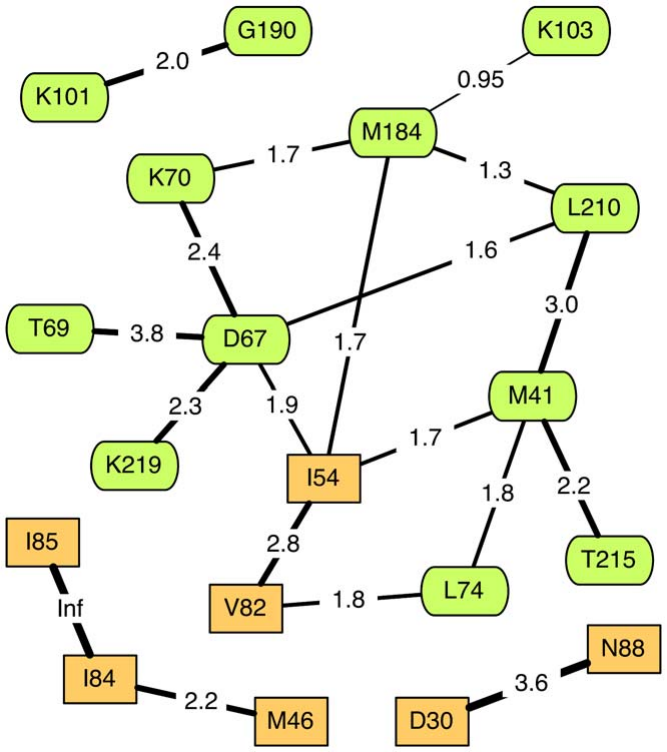
Bayesian network depicting conditional dependencies among surveillance drug resistance mutations (SDRMs) in ARV-naïve sequences. Each labeled node corresponds to a position in protease [rectangular] or RT (rounded). Nodes without dependencies (L23, L24, V32, I47, I50, F53, G73, L76, K65, V75, F77, Y115, F116, L100, V106, V179, Y181, and Y188) were omitted from the graph for clarity. Connections between nodes (edges) are labeled with the log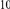 odds ratio of the 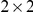 contingency table for the presence/absence of SDRMs at the respective sites, *e.g.*, an SDRM at position PR-D30 is estimated to be 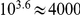 times more likely to be present in an ARV-naïve sequence that contains an SDRM at position PR-N88. Line widths for edges are also drawn in proportion to the log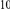 odds ratios. Inf = infinity, *i.e.*, unable to estimate because of zero count(s) in the contingency table.

Overall, 225 out of 1585 naïve sequences (14.2%) contained one or more SDRMs. Of these 225 sequences, 66 (4.2%) contained at least one PI-associated SDRM, 130 (8.2%) contained at least one NRTI-associated SDRM, and 131 (8.3%) contained at least one NNRTI-associated SDRM. In addition, 17 (1.1%) sequences contained SDRMs in all three drug classes, which is substantially greater than the number expected by chance (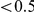 sequences) if the joint transmission of mutations was independent of drug class. A decline from pre-2003 levels in the fraction of naïve sequences with at least 1 SDRM was mirrored by a similar decline in the mean number of SDRMs per naïve sequence (Figure 1). However, we observed an increase in the most recent sample year (2008) in the upper 95% quartile of the distribution in the number of SDRMs. Neither measure of prevalence was significantly associated with demographic or risk factors in generalized linear models (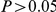). Counts of SDRMs by residue position are provided in Table 1. A Bayesian network analysis detected significant co-transmission of SDRMs in four groups (Figure 2). The largest group generally corresponded to NRTI resistance-associated mutations (with the exception of K103, I54 and V82) and contained well-characterized mutational pathways, *e.g.*, D67/T69/K70/K219 and T215/M41/L210. Two smaller groups (M46/I84/I85 and D30/N88) comprised mutations associated with PI resistance, the latter group being associated with resistance to nelfinavir in particular. The remaining group (K101/G190) comprised mutations associated with resistance to NNRTIs. In sum, co-transmitted SDRMs tended to confer resistance to the same drug or drug class.

**Table 1:** Number and percentage prevalence of putatively transmitted surveillance drug resistance mutations (SDRMs) by residue position in antiretroviral (ARV)-naïve sequences.

Class	position	count	% prevalence	Class	position	count	% prevalence
PI	L23	1	0.06	NRTI(cont'd)	K70	9	0.57
	L24	1	0.06		L74	11	0.69
	D30	15	0.95		V75	2	0.13
	V32	2	0.13		F77	1	0.06
	M46	20	1.26		Y115	1	0.06
	I47	2	0.13		F116	1	0.06
	G48	0	0		Q151	0	0
	I50	2	0.13		M184	40	2.52
	F53	1	0.06		L210	29	1.83
	I54	16	1.01		T215	77	4.86
	G73	3	0.19		K219	30	1.89
	L76	1	0.06	NNRTI	L100	1	0.06
	V82	12	0.76		K101	7	0.44
	N83	0	0		K103	82	5.17
	I84	10	0.63		V106	2	0.13
	I85	2	0.13		V179	1	0.06
	N88	15	0.95		Y181	24	1.51
	L90	30	1.89		Y188	10	0.63
NRTI	M41	49	3.09		G190	24	1.51
	K65	3	0.19		P225	1	0.06
	D67	28	1.77		M230	0	0
	T69	9	0.57				

PI = protease inhibitor; NRTI = nucleoside reverse transcriptase inhibitor; NNRTI = non-nucleoside reverse transcriptase inhibitor.

### Aggregate SDRM predictors of baseline CD4 and HIV RNA

**Figure 3:**
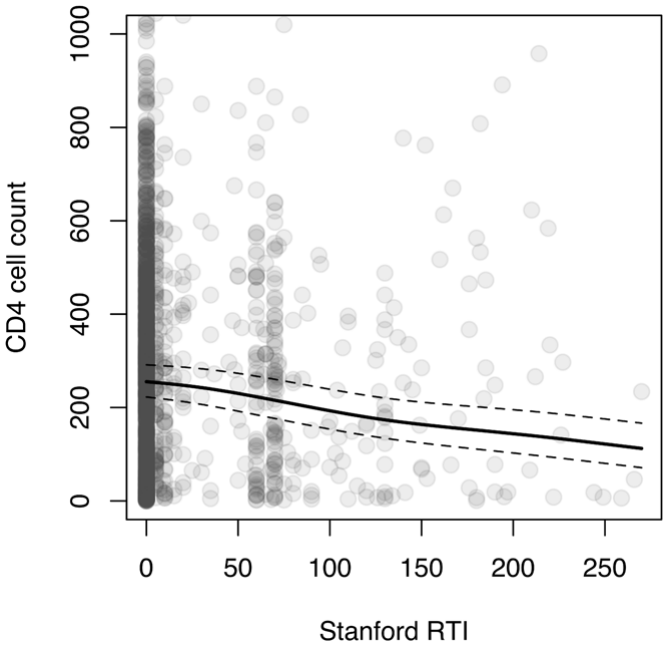
CD4 cell count (raw) plotted against the sum of Stanford scores for nucleoside and non-nucleoside reverse transcriptase inhibitors (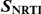 and 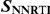, respectively). The upper limit of CD4 (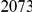 cells/mL) was truncated to 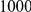 cells/mL (omitting 19 outliers) to emphasize the overall trend. Because the predicted baseline CD4 tended to decline with both 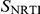 and 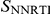, we combined the scores into a single ordinal variable to facilitate interpretation. The linear model prediction is displayed as a solid line (generated by fitting a smoothing spline to the predicted values with smoothing parameter 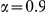), with 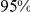 confidence intervals displayed as dashed lines.

**Figure 4:**
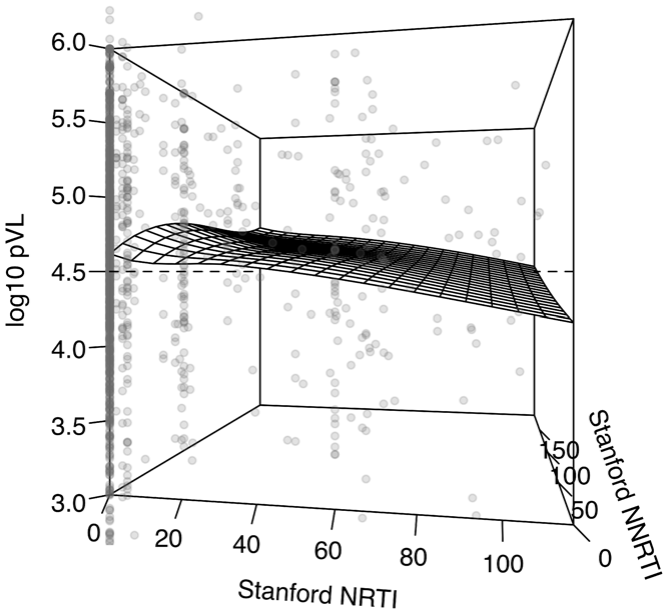
Three-dimensional scatterplot of 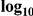-transformed plasma viral load (pVL) as a function of Stanford scores for NRTI and NNRTIs (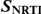 and 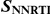). Overall, higher 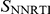 was associated with higher pVL, while 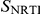 was inversely associated with pVL. The range of 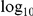 pVL (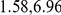) was truncated to emphasize the overall trend (omitting 73 outliers). The linear model prediction is displayed as a wireframe surface (generated by local polynomial regression with smoothing parameter 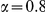).

The summated Stanford scores for NRTI and NNRTI (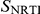, 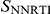) and race/ethnicity were significantly (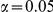) associated with baseline CD4 (Table 2). For every 10 units of 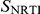 and 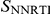, the model predicted a decline in 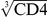 by about 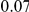 and 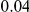 units, respectively. For example, if a hypothetical patient whose CD4 count matched the study median (285 cells/mL) became infected by an HIV-1 variant carrying SDRMs such that 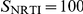 and 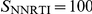, we would predict a baseline CD4 count of 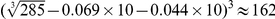 cells/mL. The joint effect of transmitted RTI resistance on CD4 is illustrated in Figure 3. Only about 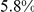 of the variance in 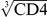 was explained by this model. Similarly, 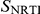, 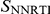, gender and race/ethnicity were significantly associated with baseline pVL (Table 2). For every 10 units in 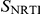, pVL was predicted to decline by about 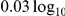 units; in contrast, pVL was predicted to increase by about 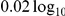 units for every 10 units in 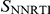 (Figure 4). Only 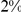 of the variance in 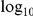 pVL was explained by this model. 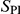 was not significantly associated with either CD4 or pVL at baseline.

**Table 2:** Coefficient estimates from Akaike Information Criterion (AIC)-selected linear models of baseline 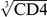 and plasma HIV RNA on Stanford scores by drug class and demographic and risk factors.

	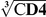		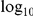 plasma HIV RNA	
	Estimate (95% CI)	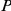 value	Estimate (95% CI)	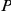 value
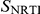	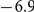 ( 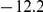 , 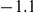 ) 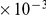	0.02	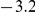 ( 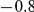 , 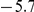 ) 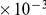	0.009
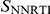	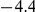 ( 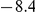 , 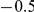 ) 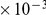	0.035	1.7 (0.04, 3.4) 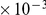	0.045
Male	NS		0.19 (0.06, 0.32)	0.006
Race/ethnicity				
Black	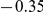 ( 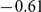 , 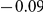 )	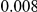	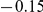 ( 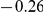 , 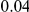 )	0.007
Hispanic	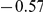 ( 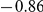 , 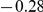 )	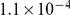	NS	

All estimates were averaged over an ensemble of linear models using Akaike weights. Only statistically significant (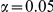) terms after weighting are reported. Linear effects of factors were estimated relative to the model intercept, *i.e.*, a hypothetical female white individual. NS = not significant.

### Position-specific SDRM predictors of baseline CD4 and HIV RNA

Although aggregate SDRM-based statistics such as Stanford scores are convenient and easy to interpret as proximate measures of drug class-specific resistance, they may mask the effects of individual mutations on clinical outcome. Consequently, we assessed the effects of position-specific SDRMs in linear models of baseline CD4 and pVL. To minimize the number of predictor variables and avoid over-fitting of the data, we limited our analysis to positions with a minimum SDRM frequency of 1% in naïve sequences (see Table 1). These positions were: M46, I54, and L90 in protease, and; M41, D67, M184, L210, T215, K219, K103, Y181, and G190 in reverse transcriptase. The presence or absence of SDRMs at each position was represented by a binary variable (herein denoted by appending an ‘X’ to the amino acid positional notation, *e.g.*, M46X = {0,1}).

**Figure 5:**
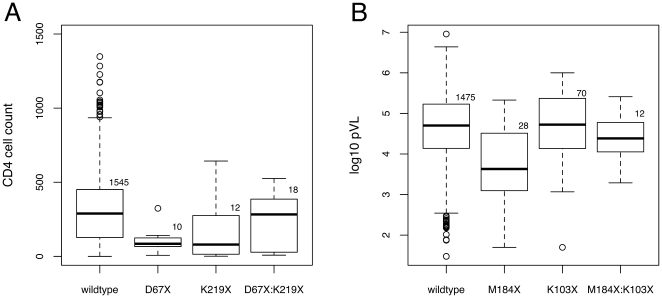
Box-and-whisker plots illustrating (A) the effects of SDRMs at positions D67 and K219 on CD4 cell count and (B) the effects of SDRMs at positions M184 and K103 on plasma viral load (pVL). ‘Wildtype’ denotes sequences lacking SDRMs at both positions, irrespective of whether any other SDRMs were present at other positions in protease or RT. Solid lines indicate the group median and open circles denote outliers that fall outside the region defined by 1.5 times the interquartile range. Plot (A) was rescaled with respect to untransformed CD4 counts to emphasize differences among groups, trimming 4 outliers from the ‘wildtype’ group (1602, 1626, 1838, and 2073 cells/mL) from the plot region. Sample sizes per group are annotated on each plot.

D67X and K219X were each associated with significantly lower baseline CD4 (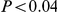; Table 3); both sets of SDRMs are thymidine analog mutations (TAMs) conferring resistance to NRTIs. For example, the model would predict a hypothetical decline from 400 cells/mL to 146 and 235 cells/mL with the introduction of SDRMs at positions D67 and K219, respectively. In addition, there was a significant interaction effect between D67X and K219X associated with a compensatory increase by about 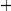
2.5 units 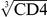 (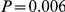; Figure 5). This model accounted for 6.8% of the variance in 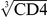.

**Table 3:** Coefficient estimates from AIC-selected linear models of baseline 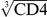 and 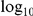 plasma HIV RNA on position-specific SDRMs and demographic and risk factors.

	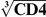		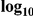 plasma HIV RNA	
	Estimate (95% CI)	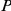 value	Estimate (95% CI)	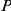 value
M41X	1.0 (0.05, 1.95)	0.04	NS	
M46X	NS		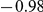 ( 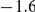 , 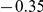 )	0.003
D67X	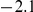 ( 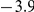 , 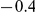 )	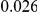	0.96 (0.25, 1.7)	0.009
M184X	NS		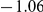 ( 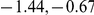 )	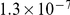
L210X	NS		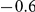 ( 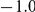 , 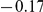 )	0.007
K219X	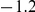 ( 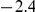 , 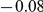 )	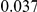	NS	
D67X:K219X	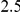 ( 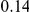 , 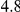 )	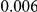	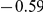 ( 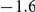 , 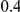 )	0.047
K219X:G190X	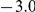 ( 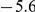 , 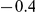 )	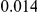	NS	
L90X:M41X	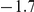 ( 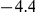 , 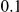 )	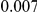	NS	
I54X:L210X	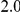 ( 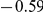 , 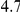 )	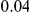	NS	
M46X:T215X	NS		1.7 (0.88, 2.5)	0.033
M184X:K103X	NS		0.58 ( 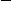 0.14, 1.3)	0.018
M184X:K219X	NS		0.87 ( 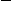 0.07, 1.8)	0.023
K219X:Y181X	NS		1.43 (0.34, 2.5)	0.013
Male	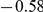 ( 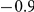 , 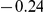 )	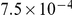	NS	
Sample date	23.3 (7.4, 38.9) 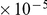	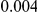	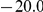 ( 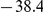 , 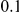 ) 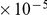	0.04
Race/ethnicity				
Black	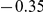 ( 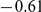 , 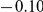 )	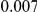	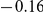 ( 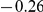 , 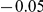 )	0.003
Hispanic	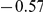 ( 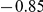 , 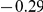 )	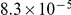	NS	
other	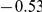 ( 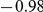 , 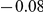 )	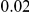	NS	

SDRMs are annotated by HXB2 reference residue and position (gene-specific numbering), followed by an ‘X’ to indicate a non-reference residue. Positions 46, 54, and 90 are in protease and all others are in reverse transcriptase. All estimates were averaged over an ensemble of linear models using Akaike weights. Only terms that were statistically significant (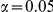) are reported here. NS = not significant.

M184X was significantly associated with an approximately ten-fold lower pVL at baseline (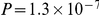; Table 3). M46X and L210X were also significantly associated with lower baseline pVL (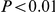) and D67X was associated with greater pVL (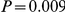). We also observed several significant mutational interaction terms in the model. For instance, interactions between M184X and either K103X or K219X were associated with a compensatory increase in pVL (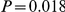 and 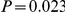, respectively; Figure 5). Similarly, a decline in pVL associated with M46X was compensated by T215X (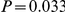). An interaction term between D67 and K219, which was encountered in the preceding model on CD4, was also statistically significant in this model of pVL (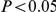) with the presence of K219X diminishing the effect of D67X on pVL by about 0.6 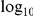 units. Approximately 6.7% of variation in pVL was explained in this model, over three times as much as the aggregate model.

### Causal inference

We used an inverse probability weighting scheme (using stabilized weights) to disentangle the causal effects of SDRMs from confounding variables that influence both the probability of the ‘treatment’ (an SDRM at the position in question) and CD4 or pVL. Based on the results of the preceding section, we focussed on the compensated and uncompensated effects of M184X on baseline pVL, and of D67X on both pVL and 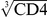. K103X and K219X were assumed to be interchangeable with respect to their compensatory interactions with M184X. Consequently, the multinomial outcome 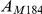 comprised 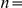
1445, 19 and 15 cases for the respective levels 0, 1 and 2 (*i.e.*, wildtype, uncompensated M184X, and M184X compensated by K103X and/or K219X). In an unweighted univariate linear model, M184X was significantly associated with a 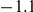 decline in 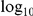 pVL in the absence of K103X and/or K219X (Table 4). Stabilized weights adjusting for the conditional probability of carrying M184X ranged from 0.02 to 18.72 with a mean of 1.016. Adding stabilized weights to the model exposed a greater deleterious effect of M184X on 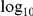 pVL (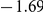 units) when uncompensated by K103X and/or K219X. Similarly, re-weighting linear models resulted in larger estimated effects of uncompensated D67X on both baseline CD4 (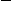
2.1 units 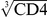) and pVL (2.5-fold increase; Table 4). Stabilized weights adjusting for the conditional probability of carrying D67X ranged from 0.006 to 12.3 with a mean of 1.003. For both pVL and CD4, the effects of D67X compensated by K219X became statistically significant and reversed sign relative to the unadjusted models (Table 4).

**Table 4:** Unadjusted and stabilized weight-adjusted linear models of multinomial exposure variables 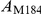 (M184X and (K103X or K219X)) and 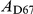 (D67X and K219X) on 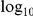 plasma HIV RNA and 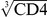, respectively.

Level	Unadjusted model		Adjusted model	
	Estimate (95% C.I.)	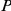	Estimate (95% C.I.)	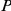
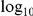 plasma HIV RNA 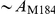				
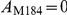	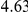 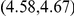			
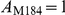	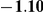 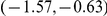	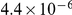	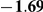 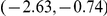	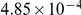
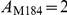	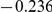 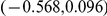	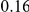	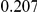 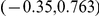	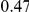
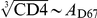				
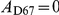	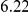 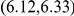			
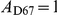	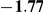 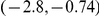	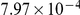	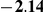 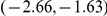	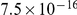
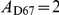	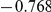 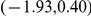	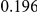	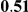 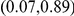	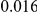
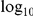 plasma HIV RNA 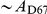				
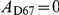	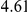 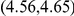			
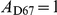	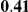 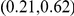	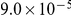	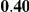 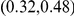	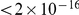
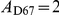	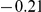 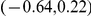	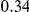	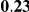 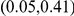	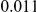

The model intercepts are taken to be 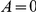 by default, *i.e.*, none of the designated mutations are present; these estimates are unchanged in the adjusted model and are not repeated for clarity of presentation. Significant (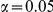) effect estimates are bolded.

## Discussion

The overall prevalence of transmitted drug resistance (TDR) mutations in therapy-naïve patients has been reported in a large number of study populations around the world and ranges from 0 to 25% [1]. On the other hand, the transmission of a specific mutation is a relatively infrequent event whose prevalence requires very large samples (over 1000 individuals) to estimate. This requirement has generally been achieved by integrating study populations from similar geographic regions, such as the Combined Analysis of Resistance Transmission over Time of Chronically and Acute Infected HIV Patients (CATCH) study encompassing 27 countries in Europe [31], the Swiss HIV Cohort Study [32], the UK Collaborative HIV cohort study [14], and the Centers for AIDS Research (CFAR) Network of Integrated Clinical Systems (CNICS) cohort reported in this study. Here we briefly compare and contrast the prevalence of drug class-specific and site-specific TDR across these integrated cohorts. First, NRTI-associated SDRMs generally tend to be more prevalent than SDRMs associated with PIs or NNRTIs. Our sample deviates slightly from this trend in that the prevalence of naïve sequences with 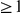 NNRTI-associated SDRM is similar to sequences with 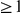 NRTI-associated SDRM, largely due to an atypically high frequency of transmitted mutations at RT-K103 (5.2%) in our sample. Second, SDRMs that are slow to revert in the absence of selection (*e.g.*, M41L, K103N, and T215X in RT and L90M in protease), implying a negligible cost to fitness [1], tend to have relatively high prevalence in therapy-naïve sequences.

The foremost and best -documented clinical implication of TDR is that it impedes the virological response to subsequent drug therapy [6]–[8]. In contrast, the clinical implications of TDR in the absence of drug therapy are subtle and less well understood. These effects have been difficult to elucidate because the transmission of SDRMs remains an infrequent event at the scale of individual mutations. Investigators have attempted to overcome the limitations of insufficient sample sizes by evaluating the effects of aggregate statistics of TDR, such as an ‘all-or-none’ statistic that groups sequences carrying one or more SDRMs [14], [16], [17]. While this approach is convenient and easy to interpret, it is subject to confounding because different drug classes or mutations can have opposite effects on prognostic clinical markers. Our use of a large data set from a multi-center cohort enabled us to overcome some limitations of sample size, and to break down the effects of TDR on baseline CD4 and pVL by drug class or amino acid position. For example, we found that 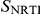 and 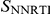 had opposing associations with pVL; because these quantities are correlated, their effects on pVL would have been confounded in an ‘all-or-none’ analysis. Similarly, we have observed several compensatory interactions among mutations that would otherwise mask the actual effects of individual mutations on CD4 or pVL (*e.g.*, D67N/G and K219Q/E).

Because this study population was comprised of seroprevalent rather than seroincident individuals , each baseline measurement of CD4 and pVL was sampled at a point in time following an unknown date of transmission. Consequently, there is no guarantee that these baseline measurements were taken at a consistent stage of infection. In other words, the CD4 count or pVL at baseline is the result of dynamics over an unknown period of time, starting from an unknown value upon infection. These missing data would be required to calculate rates of change, which would be easier to generalize to specific cases in clinical practice where these quantities (time since infection and prognostic markers at infection) may be known. This is a common problem of observational cohort studies and may explain why a substantial fraction (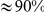) of variation in baseline CD4 or pVL remained unaccounted for in the linear models. Furthermore, because the baseline samples were taken either upon entry into care or prior to initiating ARV therapy (ART), they may constitute a biased sample of the patient population with respect to CD4 and pVL. Recent guidelines, for example, indicate that patients should be initiated on ART when their CD4 cell count falls below a threshold of 500 cells per mL regardless of HIV RNA [33] However, treatment guidelines have evolved over time [34] and using a large sample size covering a decade of clinical practice can ameliorate some of these biases. As guidelines have pushed initiation of therapy to earlier time points, it could be argued that less fit virus, with lower HIV RNA and higher CD4, would not merit clinical significance since virtually every patient should start therapy soon after identification; in other words, an association of TDR with lower viral load would not result in a longer delay between detection of HIV and the need to start therapy. However, viral fitness and replication level may be very significant in the prevalence of resistance mutations in the circulating virus population, the pool of virus in a community that could potentially be transmitted. As the pool of patients with suppressed virus increases due to ARV treatment, the relative rates of transmitted resistance may paradoxically increase due to a diminishing pool of untreated wildtype virus and a greater prevalence of resistance in the circulating virus population (10%–20% of patients who are failing therapy [1]). In this scenario, viral fitness and its effect on HIV RNA could determine which viruses are transmitted. Mutations such as M184V may be transmitted less often due to the reduced overall viral load in those subjects, whereas mutations such as K103N may increase in prevalence. THe overall rates of transmitted resistance would be a complex interplay between many host and viral factors.

Some of these issues might be resolved by the analysis of longitudinal data that are available for this study population [18]. However, this approach would create even more problems than it could potentially address. First, we would have to accommodate the effects of antiretroviral therapy that generally follows soon after HIV genotyping at baseline. This is not a trivial task, not only because it introduces a potentially large number of variables to the analysis (one for every drug, or a minimum of three for the predominant drug classes), but also because variation in adherence and confounding due to variation in prescribed regimens across clinical sites would severely complicate the interpretation of results. Second, we would have to accommodate large amounts of missing data due to differential follow-up among patients as well as a sparsity of HIV sequence data relative to CD4 and pVL measurements. In other words, HIV genotyping is performed much less frequently. Third, it is more difficult to adjust for confounding in longitudinal than cross-sectional data sets by inverse probability weighting. Previous studies have successfully employed a marginal structural modelling approach in similar but simplified contexts [20], [35]. Marginal structural models are essentially a time-dependent generalization of the inverse probability weighting approach used here [19]. However, they are difficult to apply in highly-structured contexts (where pVL, CD4, drug regimen and HIV genotype all influence one another over time) and large numbers of missing data. In sum, the analysis of longitudinal data is a substantially different question outside the scope of this study on the effect of TDR on pre-therapy pVL and CD4 and must be left to future work.

Of all position-specific effects of SDRMs on baseline prognostic markers inferred from these data, one of the largest and most statistically significant was M184V/I. The effects of these substitutions on the kinetics and fidelity of HIV-1 reverse transcriptase and replication capacity *in vitro* are well-characterized [36]–[38]. Previous studies have also documented compensatory/epistatic interactions of other mutations acting on M184V/I, including K219Q [11] and N384I [39]. Furthermore, Paredes and colleagues have recently observed that *in vivo* fitness is reduced in viruses carrying the M184V mutation in the absence of lamivudine [12]. Even so, the effect of M184V/I on viral fitness *in vivo* is confounded by the effects of other SDRMs and demographic/risk factors that should be handled using a causal modeling approach [19]. Taking such an approach, we have found that uncompensated M184V/I mutations are causally associated with a 50-fold reduction (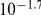) in baseline pVL in therapy-naïve patients. Previous studies have reported no greater than a 3-fold (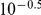) reduction in pVL due to M184V/I [38], [40], [41], while M184I itself has been estimated to reduce fitness by 23% relative to M184V [42]. This discrepancy may be due to failing to account for interactions with other mutations such as the compensatory interactions with K103 and/or K219, or confounding by other genetic and non-genetic factors affecting pVL. On the other hand, experimental studies of M184V/I-containing recombinant HIV-1 are potentially not subject to these confounding factors, and M184V has been reported to reduce fitness by as much as 16-fold *in vitro* [11]. However, it is difficult to estimate the fitness effect of a mutation *in vivo* from *in vitro* measures of its effect on replication efficiency as there are other components of RT functionality that can be affected [43].

In contrast, the clinical consequences of D67N/G and K219Q/E are not as well known. The effects of D67N/G and K219Q/E on CD4 in our study were mirrored by significant effects on pVL; *i.e.*, substitutions at D67 were causally associated with a 2.5-fold increase in pVL that was only partially compensated by substitutions at K219. These residues potentially form a salt bridge and likely affect the formation of the RT 3′ pocket during polymerization [44]. Substitutions at these sites have previously been suggested to incur a moderate fitness cost *in vitro* [11], [45], although these previous studies had not employed statistical methods to separate out the effects of these substitutions from confounding by other mutations and demographic or risk factors . Our results give the first evidence that the transmission of D67N/G may have deleterious virological and immunological consequences in therapy-naïve patients that may be compensated by K219Q/E.
